# YME1L overexpression exerts pro-tumorigenic activity in glioma by promoting Gαi1 expression and Akt activation

**DOI:** 10.1093/procel/pwac011

**Published:** 2022-10-19

**Authors:** Fang Liu, Gang Chen, Li-Na Zhou, Yin Wang, Zhi-qing Zhang, Xihu Qin, Cong Cao

**Affiliations:** Department of Neurosurgery, The Affiliated Changzhou No.2 People’s Hospital of Nanjing Medical University, Changzhou 213004, China; Department of Neurosurgery, The First Affiliated Hospital of Soochow University, Suzhou 215006, China; Department of Radiotherapy and Oncology, Affiliated Kunshan Hospital of Jiangsu University, Suzhou 215300, China; Department of Neurology and Clinical Research Center of Neurological Disease, The Second Affiliated Hospital of Soochow University, Suzhou 215004, China; Jiangsu Key Laboratory of Neuropsychiatric Diseases and Institute of Neuroscience, Soochow University, Suzhou 215031, China; Department of Neurology and Clinical Research Center of Neurological Disease, The Second Affiliated Hospital of Soochow University, Suzhou 215004, China; Jiangsu Key Laboratory of Neuropsychiatric Diseases and Institute of Neuroscience, Soochow University, Suzhou 215031, China; Department of General surgery, The Affiliated Changzhou No.2 People’s Hospital of Nanjing Medical University, Changzhou 213004, China; Department of Neurology and Clinical Research Center of Neurological Disease, The Second Affiliated Hospital of Soochow University, Suzhou 215004, China; Jiangsu Key Laboratory of Neuropsychiatric Diseases and Institute of Neuroscience, Soochow University, Suzhou 215031, China

**Dear Editor,**

Identifying novel glioma-driven signaling molecules and exploring the corresponding molecularly targeted therapies are essential for better and efficient glioma therapy. YME1L (YME1 Like 1 ATPase), a primary member of the AAA family of ATPase, is located at the inner mitochondrial membrane (Anand et al., 2014; MacVicar et al., 2019; Ohba et al., 2020). YME1L is essential for maintaining mitochondrial morphology, function, and plasticity (Anand et al., 2014; MacVicar et al., 2019; Ohba et al., 2020). YME1L assembles into a homo-oligomeric complex within the inner mitochondrial membrane (Anand et al., 2014; MacVicar et al., 2019; Ohba et al., 2020). Moreover, YME1L can degrade mitochondrial proteins, including lipid-transferring proteins, IM translocation proteins, and the dynamin-like GTPase optic atrophy 1 (OPA1) (Anand et al., 2014; MacVicar et al., 2019; Ohba et al., 2020). YME1L depletion accelerated OMA1-dependent long-form OPA1 cleavage, resulting in short-form OPA1 accumulation, increased mitochondrial fission, and mitochondrial fragmentations (Wai et al., 2015). YME1L controls the accumulation of respiratory chain subunits and is required for apoptotic resistance, cristae morphogenesis, and cell proliferation (Stiburek et al., 2012). YME1L-mediated mitochondrial reshaping is required for the growth of pancreatic ductal adenocarcinoma cells (MacVicar et al., 2019). Conversely, YME1L silencing or knockout disrupted mitochondrial functions and inhibited PDAC cell growth (MacVicar et al., 2019). Nevertheless, the expression and potential functions of YME1L in human glioma have not been studied.

Gαi proteins, or guanine nucleotide-binding protein G(i) subunit alpha, have three subunits, Gαi1, Gαi2, and Gαi3 (Fan et al., 2011). Our group has identified an essential role of Gαi proteins in transducing signals by multiple receptor tyrosine kinases (RTKs) (Cao et al., 2009; Zhang et al., 2015; Liu et al., 2018; Marshall et al., 2018; Sun et al., 2018; Bai et al., 2021; Wang et al., 2021). Gαi proteins associated with ligand-activated RTKs, required for the transduction of downstream oncogenic signalings, including phosphatidylinositol-3-kinase (PI3K)-Akt-mammalian target of rapamycin (mTOR) and extracellular signal-regulated kinase (Erk)-mitogen-activated protein kinase (MAPK) cascades (Cao et al., 2009; Zhang et al., 2015; Liu et al., 2018; Marshall et al., 2018; Sun et al., 2018; Bai et al., 2021; Wang et al., 2021). Gαi overexpression is also essential for the progression of glioma and other cancers (Liu et al., 2018; Lv et al., 2021; Wang et al., 2021).

In this study, we will show that YME1L overexpression exerts pro-tumorigenic activity in glioma by promoting Gαi1 expression and Akt activation. First, The Cancer Genome Atlas (TCGA) database was first consulted to retrieve *YME1L* RNA sequencing data in human glioma. As shown, in the human glioma tissues (“Tumor,” *n* = 166), the number of *YME1L* mRNA transcripts is significantly higher than that in the normal brain tissues (“Normal,” *n* = 1,157) (*P* < 0.001, Fig. 1A). Of the normal brain tissues, 1,152 of them were retrieved from the Genotype-Tissue Expression (GTEx) database and five tissues were from TCGA database (tumor-surrounding normal brain tissues) (Fig. 1A). The subgroup analyses based on clinical characteristics showed that high *YME1L* mRNA expression in human glioma tissues was correlated with IDH (isocitrate dehydrogenase) mutation (*P* < 0.001, Fig. 1B). It was not correlated with age (Fig. 1C) and sex (Fig. 1D) of the patients.

**Figure 1.**
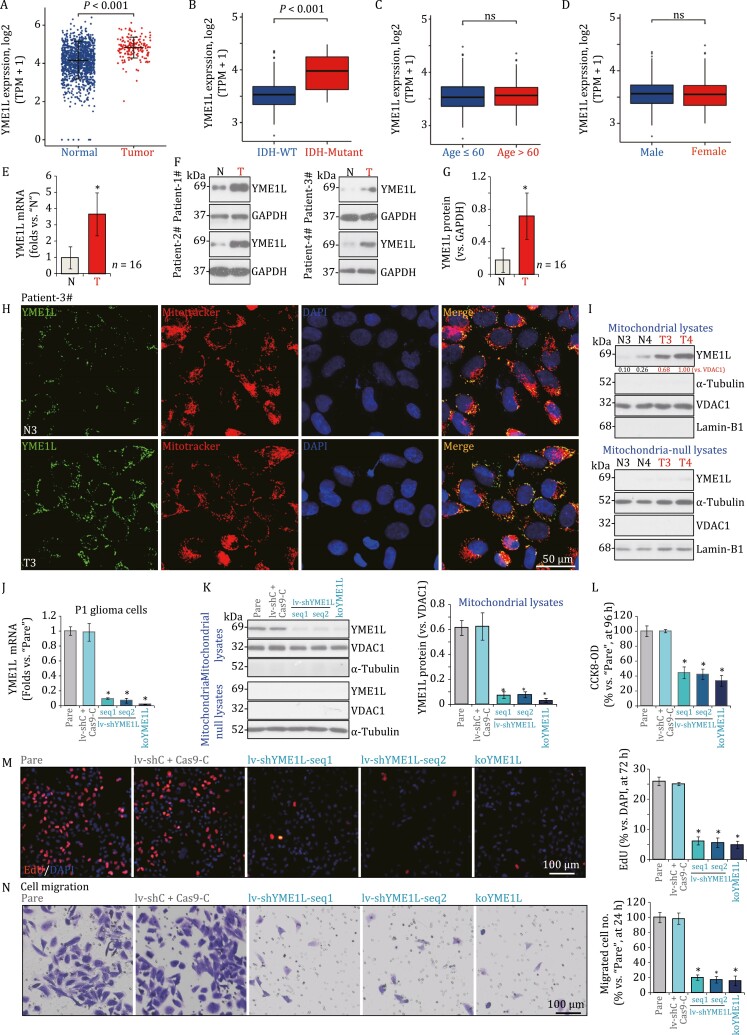
**YME1L upregulation in glioma is crucial for cell growth and migration**. (A)TCGA database shows *YME1L* expression (RNA-Seq) in glioma tissues (“Tumor”, *n* = 166) and in the normal brain tissues (“Normal”, *n* = 1,157). (B–D)The subgroup analyses of *YME1L* mRNA expression and clinical characteristics of glioma patients were shown. (E) Human glioma tissues (“T”) and the paired normal brain tissues (“N”) derived from a total of 16 HGG patients were homogenized and dissolved in the tissue lysis buffer, *YME1L mRNA* and protein expression was tested by qRT-PCR and (F–G) Western blot assays, respectively, with results quantified. (H) The human tissue immuno-fluorescence images of YME1L (green fluorescence) and the MitoTracker red in the glioma slide and the adjacent normal brain slide of one representative glioma patient (Patient 3). (I) Expression of listed proteins in mitochondrial lysates and mitochondria-null lysates of two representative glioma patients (Patients 3 and 4) was shown. (J) The P1 primary human glioma cells, stably expressing the applied YME1L shRNA (“lv-shYME1L-seq1/2”, two different sequences) or the lenti-CRSIPR/Cas9-YME1L-KO-puro construct (“koYME1L”) were established. Control P1 glioma cells were transduced with the lentiviral scramble shRNA plus the CRSIPR/Cas9 empty vector (“lv-shC + Cas9-C”). Expression of *YME1L* mRNA and (K) listed proteins (in mitochondrial lysates and mitochondria-null lysates) was shown. (L) Cells were further cultured for applied time periods, cellular functions, including cell viability (CCK-8 OD), (M) cell proliferation (EdU staining assays) and (N) cell migration were tested by the mentioned assays. “Pare” stands for the parental control cells. The data were presented as mean ± standard deviation (SD). *P < 0.05 vs. “Normal”/“N”/“Pare”. “ns” stands for non-statistical difference (*P* > 0.05). (M–N) The *in vitro* experiments were repeated five times with similar results obtained. Scale bar = 50 μm (H). Scale bar = 100 μm.

To confirm the significance of the bioinformatics observations, we tested YME1L expression in local human glioma tissues (“T”) and surrounding normal brain (“N”) tissues, from a total of 16 grade III–IV glioma (HGG) patients (see our previous studies such as Liu et al. (2018) and Wang et al. (2021). The real-time quantitative reverse transcription PCR (qRT-PCR) assay results in Fig. 1E showed that *YME1L* mRNA expression in glioma tissues was significantly higher than that in the normal tissues. Testing YME1L protein expression, using Western blot assays, further confirmed YME1L protein upregulation in glioma tissues of four representative glioma patients (Patients 1–4, Fig. 1F). Western blot quantification results further confirmed that YME1L protein upregulation is significant in glioma tissues (*P* < 0.001 vs. “N” tissues, Fig. 1G).

The tissue immuno-fluorescence images, Figs. 1H and S1A, show that YME1L protein (green fluorescence) is co-localized with the mitochondrial marker MitoTracker (red fluorescence) in both glioma slides and the adjacent normal brain slides of two representative glioma patients (Patients 3 and 4). More importantly, YME1L fluorescence intensity in the human glioma slides was significantly higher than that in the adjacent normal brain tissue (Figs. 1H and S1A).

Furthermore, an examination of mitochondrial lysates isolated from fresh human glioma tissues of four representative patients (Patients 1–4) confirmed that YME1L was enriched in the mitochondria fraction (Figs. 1I and S1B), as indicated by VDAC1 (voltage-dependent anion-selective channel 1), a mitochondrial marker protein (Figs. 1I and S1B). Lamin-B1 is a nuclear marker protein and α-tubulin is a cytosol marker protein (Figs. 1I and S1B). Once again, mitochondrial YME1L protein expression in glioma tissues was significantly elevated (Figs. 1I and S1B). Conversely, YME1L protein expression was almost not detected in the mitochondria-null lysates of human tissues (Figs. 1I and S1B). These results show that YME1L protein is upregulated and localized to the mitochondria of glioma tissues.

YME1L expression in the glioma cells was tested next. The established glioma cell lines, A172 and U251, as well as the primary human glioma cells that were derived from three different patients, “P1”, “P2”, and “P3” (Wang et al., 2021), were tested. *YME1L* mRNA expression in the glioma cells was significantly higher than that in the primary human astrocytes (Fig. S1C). Moreover, YME1L protein upregulation was detected in the immortalized and primary glioma cells (Fig. S1D). Whereas in the primary astrocytes, YME1L protein expression is low (Fig. S1D). These results together showed that YME1L is upregulated in human glioma.

To silence YME1L expression, the P1 primary human glioma cells (Liu et al., 2018; Wang et al., 2021) were individually transduced with two different lentiviral YME1L small hairpin RNA (shRNA) (lv-shYME1L-seq1 and lv-shYME1L-seq2, with different sequences). Stable cells were established following selection by puromycin. Alternatively, the clustered regularly interspaced short palindromic repeats (CRISPR)/CRISPR-associated protein 9 (Cas9) method was utilized. A lenti-CRSIPR/Cas9-YME1L-knockout (KO)-puro construct was transduced to the P1 glioma cells. Single stable cells were established by puromycin selection and YME1L KO screening, namely koYME1L cells. As compared to control P1 glioma cells with the lentiviral scramble shRNA (shC) plus the CRSIPR/Cas9 empty vector (“lv-shC + Cas9-C”), *YME1L* mRNA expression was dramatically downregulated in shYME1L-expressing cells and koYME1L cells (Fig. 1J). Western blot testing the mitochondrial fraction lysates confirmed specific depletion of YME1L protein in the mitochondria of P1 human glioma cells by the applied shRNA and KO strategies (Fig. 1K). While its expression was not detected in mitochondria-null lysates of P1 glioma cells with or without the applied genetic treatments (Fig. 1K).

As shown, the cell counting kit-8 (CCK-8) optical density (OD) was dramatically decreased in YME1L-silenced or koYME1L P1 glioma cells (Fig. 1L), indicating viability reduction. In addition, Fig. 1M showed that 5-ethynyl-2ʹ-deoxyuridine (EdU)-positive nuclei ratio was dramatically decreased in YME1L-silenced and koYME1L glioma cells, supporting the anti-proliferative activity by YME1L depletion in primary glioma cells. “Transwell” assay results showed that YME1L silencing or KO potently inhibited P1 glioma cell migration (Fig. 1N). Unsurprisingly, the control lv-shC+Cas9-C treatment failed to significantly affect P1 glioma cell functions (Fig. 1L–N).

The primary human glioma cells that were derived from two other patients [“P2” and “P3” (Liu et al., 2018; Wang et al., 2021)] as well as the immortalized cell lines (A172 and U251) were cultured and infected with lv-shYME1L-seq1-expressing lentivirus. Stable cells were again established by puromycin selection. The qRT-PCR assay results, Fig. S1E, confirmed that *YME1L* mRNA levels were robustly decreased in the glioma cells with YME1L shRNA. shRNA-induced silencing of YME1L largely inhibited the viability (CCK-8 OD) of the primary and established glioma cells (Fig. S1F). Moreover, cell proliferation, tested by the EdU-positive nuclei ratio (Fig. S1G), and *in vitro* cell migration (“Transwell” assays, Fig. S1H) were potently inhibited by the YME1L shRNA. Together, YME1L silencing or KO resulted in significant anti-glioma cell activity, inhibiting cell survival, proliferation, and migration.

Whether YME1L depletion could provoke apoptosis activation in glioma cells was studied next. As shown, the caspase-3 activity and the caspase-9 activity were both significantly increased in the YME1L-silenced or the koYME1L P1 glioma cells (Fig. S2A). Figure S2B showed that YME1L shRNA or KO resulted cleavages of caspase-3 and poly(ADP-ribose) polymerase (PARP) in P1 glioma cells. In addition, the histone-associated DNA fragments were robustly increased in P1 glioma cells after YME1L silencing or KO (Fig. S2C). The terminal deoxynucleotidyl transferase dUTP nick end labeling (TUNEL)-positive nuclei ratio was significantly increased in P1 glioma cells with YME1L depletion (Fig. S2D). As expected, the lentiviral scramble shRNA plus the CRSIPR/Cas9 empty vector (“lv-shC + Cas9-C”) did not induce significant apoptosis activation in P1 glioma cells (Fig. S2A–D).

In other primary human glioma cells (P2 and P3) and immortalized cell lines (A172 and U251), YME1L silencing by lv-shYME1L-seq1 (see Fig. 1) similarly induced caspase-3 activation (Fig. S2E) and cell apoptosis (evidenced by nuclear TUNEL ratio increase, Fig. S2F). In the primary human astrocytes, infection with the lv-shYME1L-seq1 resulted in robust YME1L silencing as well (Fig. S2G). Yet, the cell viability (tested by the CCK-8 OD, Fig. S2H) and cell apoptosis (tested by the TUNEL-positive nuclei ratio, Fig. S2I) were not significantly affected by YME1L silencing in the astrocytes. These results implied a glioma cell-specific effect by YME1L depletion.

YME1L is essential for maintaining mitochondrial morphology and functions. We therefore analyzed whether YME1L depletion can affect mitochondrial functions in glioma cells. In the YME1L-silenced or the koYME1L P1 glioma cells, the tetraethylbenzimidazolylcarbocyanine iodide (JC-1) color changed from orange (JC-1 aggregates) to green (JC-1 monomers) (Fig. S3A), indicating mitochondrial depolarization. In addition, the CellROX intensity was significantly increased in YME1L-depleted P1 glioma cells (Fig. S3B), suggesting that mitochondria-derived reactive oxygen species (ROS) contents were significantly increased with YME1L silencing or KO. The accumulation of single-strand DNA (ssDNA, ELISA assays) was detected as well in the YME1L-silenced/-KO P1 glioma cells, indicating DNA breaks accumulation (Fig. S3C). Moreover, following YME1L depletion, the lipid peroxidation levels were significantly augmented in P1 glioma cells (Fig. S3D), evidenced by the increased thiobarbituric acid reactive substances (TBAR) activity. In other primary human glioma cells (P2 and P3) and immortalized cell lines (A172 and U251), lv-shYME1L-seq1-induced YME1L silencing similarly induced mitochondrial depolarization and ROS production, causing JC-1 green monomer accumulation (Fig. S3E) and the CellROX intensity increase (Fig. S3F).

We next hypothesized that further increasing YME1L expression should exert cancer-promoting activity in glioma cells. The lentivirus encoding the wild-type YME1L cDNA was transfected to P1 glioma cells. Puromycin was then utilized to select stable cells: OE-YME1L-sL1 and OE-YME1L-sL2 (two lines). Fig. S4A confirmed that the *YME1L* mRNA expression was significantly elevated in OE-YME1L P1 glioma cells (versus vector control cells/“Vec”). In addition, significant YME1L protein elevation in total cell lysates was detected (Fig. S4B). YME1L protein upregulation was detected only in the mitochondria of P1 glioma cells with the YME1L-overexpressing lentiviral construct (Fig. S4B). Again no YME1L protein expression was detected in the mitochondria-null lysates (Fig. S4B).

Evidenced by the increased EdU-positive nuclei ratio, we demonstrated that ectopic overexpression of YME1L promoted P1 glioma cell proliferation (Fig. S4C). The cell vaiblity, CCK-8 OD, was enhanced as well (Fig. S4D). In addition, the number of migrated cells (Fig. S4E) was significantly enhanced in OE-YME1L glioma cells. In other primary human glioma cells (P2 and P3) and immortalized cell lines (A172 and U251), infection of the YME1L-expressing lentivirus (“OE-YME1L”) significantly increased *YME1L* mRNA expression (Fig. S4F). As a result, cell proliferation (by measuring the EdU-positive nuclei ratio, Fig. S4G) and migration (quantified results in “Transwell” assays, Fig. S4H) were augmented.

Next, TCGA database results were retrieved and differentially expressed gene (DEGs) analyses were performed to examine co-expression genes with *YME1L* in glioma tissues. By employing the Pearson Correlation Coeffcient analyses, the co-expression volcano map was shown (Fig. 2A). The top 20 DEGs that were upregulated in YME1L-high glioma tissues were presented (Fig. 2B). One key gene is *GNAI1* (encoding Gαi1 protein, Fig. 2B). Our previous studies have shown that Gαi1 associated with multiple RTKs in human glioma, required for downstream Akt activation and glioma tumorigenesis (Liu et al., 2018; Wang et al., 2021). Conversely, Gαi1 silencing, knockout, or mutation largely inhibited glioma cell growth (Liu et al., 2018; Wang et al., 2021).

**Figure 2.**
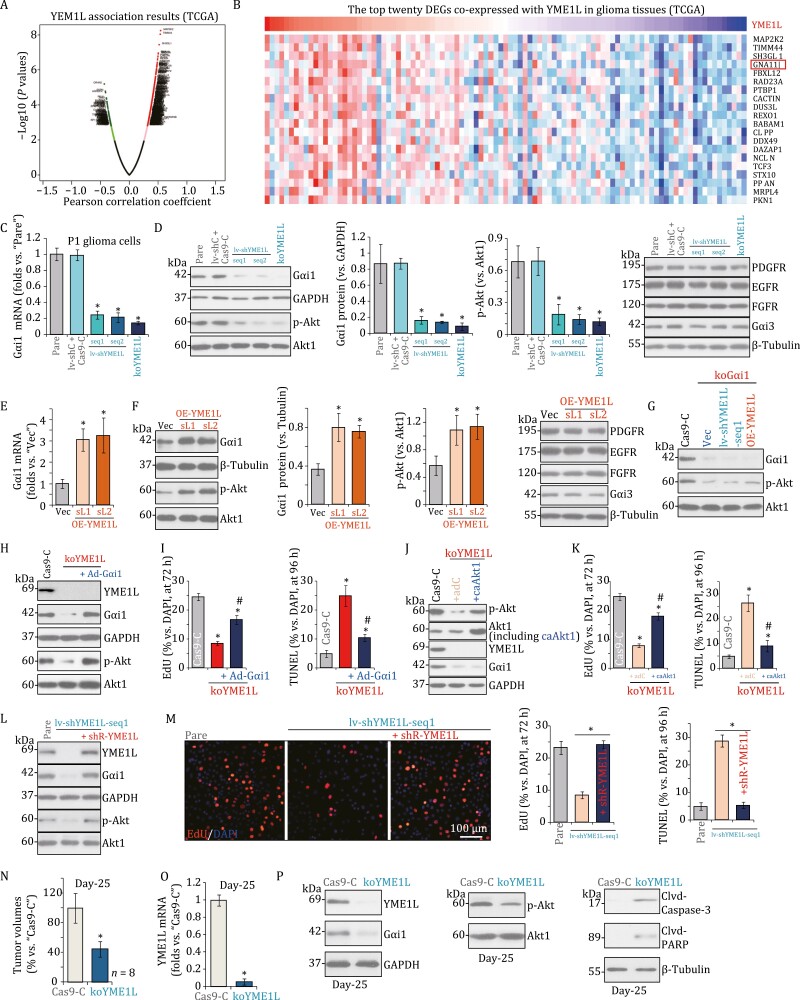
**YME1L is important for Gαi1 expression, Akt activation, and orthotopic growth of primary glioma xenografts in nude mice.** (A) The volcano map of differentially expressed gene (DEGs) based on YME1L expression in TCGA glioma tissues was shown. (B) The top 20 upregulated DEGs in YME1L-high TCGA glioma tissues were shown, *Gαi1* (*GNAI1*) was marked in the red box. (C–D) P1 glioma cells, stably expressing the applied YME1L shRNA (lv-shYME1L-seq1/2, two different sequences) or the lenti-CRSIPR/Cas9-YME1L-KO-puro construct (“koYME1L”), were established. Control P1 glioma cells were transduced with the lentiviral scramble shRNA plus the CRSIPR/Cas9 empty vector (“lv-shC + Cas9-C”), expression of listed genes and proteins (in total cell lysates) was shown. (E–F) P1 glioma cells expressing the wild-type YME1L cDNA (“OE-YME1L-sL1 or OE-YME1L-sL2”) or the empty vector (“Vec”) were established and expression of listed genes was shown. (G) P1 glioma cells expressing the lenti-CRSIPR/Cas9-Gαi1-KO construct (“koGαi1”) were further infected with lv-shYME1L-seq1 lentivirus, YME1L-expressing lentivirus (“OE-YME1L”) or the empty vector (“Vec”), stable cells were established and expression of listed proteins was shown. (H) The koYME1L P1 glioma cells were further infected with or without the adenoviral Gαi1-expresssiing construct (+Ad-Gαi1), expression of listed proteins was tested; (I) cells were further cultured for applied time periods, cell proliferation and apoptosis were tested by nuclear EdU staining and TUNEL staining assays respectively, with results quantified. (J) The koYME1L P1 glioma cells were further infected with the constitutively active Akt1 (S473D) adenovirus (“+caAkt1”) or the adenovirus with the control vector (“+adC”), expression of listed proteins was tested by Western blot assays; (K) cells were further cultured for applied time periods, cell proliferation, and apoptosis were tested similarly. (L) The P1 primary human glioma cells expressing the lv-shYME1L-seq1 were further transduced with the shRNA-resistant-YME1L (“shR-YME1L”), and stable cells established; control cells were the parental control cells (“Pare”), expression of listed proteins was shown; (M) cell were further cultured for applied time periods, and cell proliferation and apoptosis were examined similarly. The exact same amount of P1 primary human glioma cells (5 × 10^5^ cells of each mouse), expressing the lenti-CRSIPR/Cas9-YME1L-KO-puro construct (koYME1L) or control construct (“Cas9-C”), were intracranially injected to brains of nude mice; (N) after 25 days (“Day 25”), animals were decapitated and tumors were isolated by surgery and the tumor volumes were recorded. Expression of listed genes was tested by (O) qRT-PCR and (P) Western blot assays. The data were presented as mean ± standard deviation (SD). (C–F) “Pare” stands for the parental control cells. “Vec” stands for the vector control. *P < 0.05 vs. “Pare”/“Vec” cells. (I–K) *P < 0.05 vs. “Cas9-C” cells. *P < 0.05 vs. “koYME1L” cells. (M) *P < 0.05. (N–O) *P < 0.05 vs. “Cas9-C” groups. (M) The *in vitro* experiments were repeated five times with similar results obtained. Scale bar = 100 μm.

We therefore, tested whether YME1L is important for Gαi1 expression in glioma cells. As shown, shRNA-induced silencing or CRISPR/Cas9-induced KO of YME1L led to dramatic *Gαi1* mRNA (Fig. 2C) and protein (Fig. 2D) downregulation in P1 primary glioma cells. Moreover, Akt activation, tested by p-Akt, was inhibited with YME1L silencing or KO (Fig. 2D). Expression of RTKs, including epidermal growth factor receptor (EGFR), fibroblast growth factor receptor (FGFR), platelet-derived growth factor receptor α (PDGFR α) as well as Gαi3 was not significantly changed in P1 glioma cells with YEM1L shRNA/KO (Fig. 2D). Conversely, in the YME1L-overexpresed P1 glioma cells (OE-YME1L-sL1 and OE-YME1L-sL2, see Fig. S4), *Gαi1* mRNA (Fig. 2E) and protein (Fig. 2F) levels were significantly increased, and Akt activation was augmented (Fig. 2F). RTKs (EGFR, PDGFRα, and FGFR) and Gαi3 expression were again unchaged (Fig. 2F). These results implied that YME1L is vital for Gαi1 expression and Akt activation in glioma cells.

In line with our previous findings (Liu et al., 2018; Wang et al., 2021), Gαi1 KO, using the CRISPR/Cas9 method, inhibited Akt activation in P1 glioma cells (Fig. 2G). Significantly, in the Gαi1 KO cells, altering YME1L expression by the lv-shYME1L-seq1 (Fig. 1) or the lentiviral YME1L-expressing construct (OE-YME1L, see Fig. S4) failed to further affect Akt activation (Fig. 2G). These results suggested that YME1L-mediated Akt activation was due to regulating Gαi1 expression.

To test whether YME1L-driven glioma cell growth was due to mediating Gαi1-Akt signaling, the recombinant adenovirus encoding the full-length Gαi1 (“Ad-Gαi1,” no tag) was stably transduced to koYME1L P1 glioma cells, that completely restored Gαi1 expression and Akt activation (Fig. 2H). Significantly, YME1L KO-induced proliferation arrest (by measuring the EdU-positive nuclei ratio, Fig. 2I) and apoptosis activation (TUNEL staining assays, Fig. 2I) were largely attenuated by Ad-Gαi1.

Next, the constitutively active Akt1 (S473D, caAkt1) adenovirus was stably transduced to the koYME1L P1 glioma cells, and it restored Akt activation without affecting YME1L-Gαi1 expression (Fig. 2J). Significantly, caAkt1 largely inhibited YME1L KO-induced proliferation arrest (Fig. 2K) and apoptosis activation (Fig. 2K) in P1 glioma cells. These results together implied that YME1L-driven glioma cell progression was due to, at least in part, by mediating Gαi1-Akt signaling.

Importantly, transduction of a shRNA-resistant-YME1L (“shR-YME1L”) restored YME1L and Gαi1 expression as well as the Akt phosphorylation in P1 glioma cells expressing the lv-shYME1L-seq1 (Fig. 2L). YME1L shRNA-induced cell proliferation inhibition and apoptosis were significantly mitigated by the shR-YME1L in P1 glioma cells (Fig. 2M).

To study the potential effect of YME1L on glioma cell growth *in vivo*, the P1 glioma cells were *s.c.* injected to the nude mice. Within 3 weeks of cell inoculation, P1 glioma xenografts were established (“Day-0,” with tumor volume close to 100 mm^3^). The xenograft-bearing nude mice were then randomly assigned into three groups and were subject to intratumoral injection of adeno-associated virus (aav)-packed shRNA, including aav-shYME1L-seq1, aav-shYME1L-seq2 or aav-shC. The aav injection was performed daily for 14 consecutive days. The tumor growth curve results, recording tumor volumes every 6 days (“Day-0” to “Day-42”), showed that injection of shYME1L aav potently inhibited P1 glioma xenograft growth in nude mice (Fig. S5A). The volumes of aav-shYME1L-injected tumors were significantly lower than those with aav-shC injection (Fig. S5A). The estimated daily tumor growth was calculated and the following formula was utilized: (Tumor volume at “Day-42” − Tumor volume at “Day-0”)/42. Results showed that P1 glioma xenograft growth was largely inhibited after injection aav-shYME1L (Fig. S5B). P1 glioma xenografts were all isolated and weighted at “Day-42.”. We found that aav-shYME1L-injected xenografts were significantly lighter than aav-shC-injected xenografts (Fig. S5C). The mice body weights, on the other hand, were not significantly different between the three groups (Fig. S5D).

At experimental “Day-5” and “Day-10,” 3 h after the aav injection, one mouse in each group was killed after anesthesia, and tumor resections were performed. A total of six glioma xenografts were obtained and tumor lysates were tested. As shown, *YME1L* mRNA levels were dramatically decreased in the aav-shYME1L-injected tumors (Fig. S5E). YME1L protein silencing was detected as well (Fig. S5F). In addition, levels of Gαi1 and p-Akt were decreased in YME1L-silenced xenografts (Fig. S5F). Therefore, in line with the *in vitro* signaling findings, aav-shRNA-induced silencing of YME1L inhibited Gαi1 expression and Akt activation in P1 glioma xenografts. On the contrast, levels of cleaved-caspase-3 and cleaved-PARP were increased in YME1L-silenced xenografts (Fig. S5G), indicating apoptosis activation.

Next, the control P1 glioma cells with the lenti-CRSIPR/Cas9-YME1L-KO-puro construct (“koYME1L”) or control construct (“Cas9-C”), were intracranially (using the described parameters (Liu et al., 2018)) injected into brains of nude mice. At experimental day 25 (“Day-25”), when the first mouse in the Cas9-C group exhibited obvious symptoms, all mice were sacrificed and tumors were isolated (Liu et al., 2018). As shown, the volumes of the koYME1L glioma xenografts were significantly lower than those of the Cas9-C xenografts (Fig. 2N). Tumor lysates were then tested. The qRT-PCR assay results found that *YME1L* mRNA levels were significantly decreased in koYME1L glioma xenografts (Fig. 2O). YME1L protein levels were decreased as well (Fig. 2P). In addition, Gαi1 expression and Akt activation were decreased in the koYME1L glioma xenografts (Fig. 2P), and cleaved-caspase-3 and cleaved-PARP increased (Fig. 2P). Together, YME1L depletion inhibited subcutaneous and orthotopic growth of primary glioma xenografts in nude mice.

Srinivasainagendra et al. (2017) have reported that *YME1L* is frequently mutated in human colorectal cancer along with other cancers to a lesser degree. Inhibition of YME1L could promote cancer cell death both *in vitro* and *in vivo* (Stiburek et al., 2012; Wai et al., 2015). YME1L silencing led to accumulation of non-assembled respiratory chain subunits, impaired cell proliferation, altered morphology, diminished rotenone-sensitive respiration, and increased sensitivity to oxidative damage (Stiburek et al., 2012). Here we supported that YME1L overexpression exerted significant pro-tumorigenic activity and should be an important therapeutic target of glioma.

Our previous studies have supported a key role of Gαi proteins in mediating signaling by multiple RTKs and non-RTK receptors. Gαi1/3 is associated with EGF-stimulated EGFR and the adaptor protein Grb2-associated binder 1 (Gab1) to transduce downstream Akt-mTOR activation (Cao et al., 2009). Gαi1/3 are key signaling proteins of vascular endothelial growth factor (VEGF) signaling and are essential for VEGF-induced VEGF receptor 2 (VEGFR2) endocytosis, downstream signaling transduction and angiogenesis (Sun et al., 2018). In addition, Gαi1/3 silencing or KO largely inhibited BDNF-induced TrkB endocytosis and downstream Akt-mTORC1 and Erk-MAPK signaling (Marshall et al., 2018). Moreover, KGF (keratinocyte growth factor) and FGF-induced signalings also required Gαi1/3 (Zhang et al., 2015). Interestingly, a very recent study by our group found that interleukin-4-induced downstream Akt-mTOR activation and macrophage M2 polarization also required Gαi1/3 (Bai et al., 2021).

We have previously shown that *Gαi1* mRNA and protein expression was significantly elevated in human glioma tissues, being more dramatic in high-grade gliomas (Liu et al., 2018). Overexpressed Gαi1 is associated with multiple RTKs, required for downstream Akt activation and glioma cell growth (Liu et al., 2018). Conversely, *Gαi1* shRNA, dominant negative mutant interference, complete KO, or expressing the anti-Gαi1 miR-200a inhibited Akt activation and glioma cell growth (Liu et al., 2018). Moreover, Gαi1/3 mediation of neuroligin-3-induced downstream signaling is essential for neuronal-driven glioma intracranial growth (Wang et al., 2021). These results verified that Gαi1 should be an important therapeutic target of human glioma.

YME1L is important for Gαi1 expression in glioma cells. Gαi1 expression and downstream Akt activation were decreased after YME1L silencing or KO, but were augmented with YME1L overexpression in primary glioma cells. *In vivo*, Gαi1 expression and Akt activation were largely inhibited in YME1L-silenced or YME1L-KO glioma xenograft tissues. Importantly, Gαi1 re-expression, by Ad-Gαi1, restored Akt activation and largely inhibited YME1L KO-induced anti-glioma cell activity. In addition, restoring Akt activation, by caAkt1, also alleviated YME1L KO-induced proliferation inhibition and apoptosis in glioma cells. These results clearly supported that YME1L-driven glioma cell progression is mediated, at least in part, by mediating Gαi1-Akt cascade. The underlying signaling mechanisms warrant further cauterizations. In conclusion, by promoting Gαi1 expression and Akt activation, YME1L overexpression exerts significant pro-tumorigenic activity in glioma. YME1L should be an important therapeutic target of human glioma.

## Supplementary Material

pwac011_suppl_Supplementary_Data

## Footnotes

This work was generously supported by Key Research and Development Program of Jiangsu Province (No. BE2019652), Changzhou international cooperation program (CZ20200039), Development Program of Changzhou City (CE20205024) and National Natural Science Foundation of China (Grant Nos. 81922025, 81974388, 82171461 and 81771457). A Project Funded by the Priority Academic Program Development of Jiangsu Higher Education Institutions.

The authors declare that they have no competing interests. All authors conceived, designed, and supervised the study. All authors collected samples, performed the experiments and analyzed the data, and involved in drafting the article and revising it critically for important intellectual content.
