# Supplementary material for: YME1L overexpression exerts pro-tumorigenic activity in glioma by promoting Gαi1 expression and Akt activation
Source: Protein Cell. 2022 Oct 19;14(3):223–9. doi: 10.1093/procel/pwac011 (PMC10098041; doi:10.1093/procel/pwac011)
Supplement: pwac011_suppl_Supplementary_Data [file pwac011_suppl_supplementary_data.pdf]

Supplementary information

YME1L overexpression exerts pro-tumorigenic activity in glioma by promoting Gαi1 expression and Akt activation

Fang Liu [1#], Gang Chen [2#], Li-Na Zhou [3#], Yin Wang [4, 5#], Zhi-qing Zhang [4, 5], Xihu Qin [6*] and Cong Cao [4, 5*]

This file includes:
Materials and methods
Figures S1-S5

Materials and methods

Reagents. Polybrene and puromycin were purchased from Sigma-Aldrich (St. Louis, MO). The antibodies were provided by Cell Signaling Technology (Beverly, MA) and Abcam (Cambridge, UK). Fetal bovine serum (FBS), Dulbecco's modified Eagle's medium (DMEM), MEM and antibiotics were provided by Gibco-BRL (Suzhou, China). MitoTracker Red and other fluorescence dyes, TRIzol and other RNA assay reagents were purchased from Thermo-Fisher Invitrogen (Shanghai, China).

Cell culture. The established glioma cell lines, A172 and U251, were obtained from the Cell Bank of the Shanghai Biology Institute, Chinese Academy of Science (Shanghai, China). The primary human glioma cells that were derived from three written informed-consent primary glioma patients, "P1", "P2" and "P3", as well as the primary human astrocytes were reported early (Cai et al., 2017; Liu et al., 2018; Shao et al., 2018; Wang et al., 2021). Mycoplasma-microbial contamination examination, STR profiling, population doubling time and morphology were regularly checked to confirm the genotypes. Primary human glioma cells and astrocytes were cultured as described previously (Cai et al., 2017; Liu et al., 2018; Shao et al., 2018). The protocols of using human cells, according to Declaration of Helsinki, were approved by the Ethics Board of Soochow University.

Human tissues. A total of sixteen (16) high grade glioma (HGG) tissues ("T") and matched adjacent normal brain tissues ("N") were reported early (Liu et al., 2018; Shao et al., 2018; Wang et al., 2021). Tissues were derived from stage III-IV HGG patients, stored in liquid nitrogen and homogenized in tissue lysis buffer with proteasome inhibitors before further analyses. For the immunofluorescence studies, the human tissue sections (4 μm in thickness) were incubated with anti-YME1L antibody for overnight at 4 °C and the green fluorescence secondary antibody 37 °C for 60 minutes. After the mitochondria were stained by MitoTracker red (Invitrogen, Shanghai, China), the fluorescence signals were observed under confocal fluorescence microscope (Zeiss). The written-informed consent was obtained from each patient. The protocols of using human tissues were in according to Declaration of Helsinki and were approved by the Ethics Board of Soochow University.

YEM1L shRNA or overexpression. A set of two different YEM1L shRNAs with non-overlapping sequences were designed and synthesized by Genechem (Shanghai, China), and were individually sub-cloned into the GV369 construct (Genepharm). The full-length YME1L sequence or shRNA resistant YME1L, synthesized by Genechem, were individually sub-cloned into the GV369 lentiviral vector (Genechem). The construct and the lentivirus packaging plasmids (Genechem) were co-transfected into HEK-293 cells to generate YEM1L shRNA/YEM1L-expressing lentivirus. The virus was filtered, enriched (at MOI=20) and added to cells cultivated in polybrene-containing complete medium. Afterwards, cells were cultured in puromycin (2.5 μg/mL)-containing complete medium for another 96h. YEM1L knockdown or overexpression in the stable cells was confirmed by Western blotting and qRT-PCR assays. Control cells were infected with scramble shRNA lentivirus ("shC")

(Genechem) or empty vector ("Vec") (Genechem). For the *in vivo* studies, the YME1L shRNA (-seq1) or shC was sub-cloned into an adeno-associated virus (AAV) construct (AAV9, Genechem). The construct was transfected to HEK-293 cells to generate shRNA-expressing AAV. The virus was then filtered, enriched and injected to xenograft tumors.

YEM1L knockout (KO). Glioma cells were seeded into six-well plates at 60% confluence and were transfected with a Cas9-expressing construct (Genechem) by Lipofectamine 2000. Stable cells were established by puromycin selection. The sequence encoding small-guide (sgRNA) targeting YEM1L (Target DNA Sequence: *ATGATGTCGATACAAGCAAG*, PAM Sequence: *AGG*) was inserted into the lenti-CRISPR/Cas9-KO-puro construct (Genechem). The construct, lenti-CRSIPR/Cas9-YME1L-KO-puro construct, was transduced to Cas9-expressing glioma cells for 48h. Cells were then cultured in puromycin (2.5 μg/mL)-containing complete medium (for another 48h), distributed into 192-well plates and subject to YEM1L KO screening. Single stable YEM1L KO cells were then established. Control cells were transduced with the lenti-CRISPR/Cas9-KO-puro empty vector ("Cas9-C").

Cell viability. Cell viability was examined by using an Cell Counting Kit-8 (CCK-8) (Dojindo Molecular Technologies, Inc.). Briefly, cells were seeded in poly-L-lysine-coated 96-well microplates at 3×10^3 cells/well. After incubation for 96h, CCK-8 solution was added to each well for 2h at 37°C, and the absorbance of each well measured at 450 nm using a microplate reader.

TUNEL (terminal deoxynucleotidyl transferase dUTP nick end labeling) staining. Cells were seeded onto a cover slide in a 24-well plate at 4×10^4 cells/well. After indicated time periods, cells were fixed with 4% paraformaldehyde, incubated with 0.3% Triton X-100, and incubated with the TUNEL solution for 2h at 37°C. Cells were then stained with 4',6-diamidino-2-phenylindole (DAPI) for 10 min and cell nuclei visualized under a confocal microscope (Zeiss, Shanghai, China).

Other assays, including Western blotting, qRT-PCR, the nuclear EdU (5-ethynyl-20-deoxyuridine) staining assaying of cell proliferation, "Transwell" assays, and single strand DNA (ssDNA) ELISA, as well as reactive oxygen species (ROS) detection by the CellROX fluorescence dye assay, the thiobarbituric acid reactant (TBAR) activity assaying of lipid peroxidation and the mitochondrial depolarization detection by the JC-1 dye assay were described in detail previously (Cao et al., 2009; Zhang et al., 2015; Sun et al., 2018; Bai et al., 2021; Gao et al., 2021; Liang et al., 2021; Shan et al., 2021). The Histone DNA ELIA protocol was reported early (Liang et al., 2019). For all EdU staining assays, five random microscopy views were included to calculate the average EdU ratios (% versus DAPI). The same set of lysates were run in parallel gels to test different proteins. An ImageJ software was employed for the quantification of the protein band.

CRISPR/Cas9-induced Gαi1 knockout (KO). The primary human glioma cells were seeded into six-well plates at 50-60% confluence and transfected with the Cas9-expressing

construct (Genechem, Shanghai, China). Stable Cas9-expressing cells were then established after puromycin selection. A lenti-CRISPR/Cas-9 Gαi1 KO construct (see our previous studies (Sun et al., 2018; Wang et al., 2021)) was transduced to the Cas9-expressing glioma cells, and stable cells again selected by puromycin (for 96h). Gαi1 KO screening was then performed to establish single stable Gαi1 KO cells. Control cells were transduced with the lenti-CRISPR/Cas-9 empty vector ("Cas9-C") (Sun et al., 2018; Wang et al., 2021).

Gαi1 overexpression. The recombinant adenovirus containing full-length Gαi1 cDNA ("Ad-Gαi1", no tag) was described in our previous studies (Sun et al., 2018; Wang et al., 2021). The primary human glioma cells were seeded into six-well plates at 50-60% confluence and infected with the Ad-Gαi1. Stable cells were established after puromycin selection (for 72h). Gαi1 expression was verified by qRT-PCR and Western blotting assays.

Constitutively-active mutant Akt1. The primary human glioma cells were seeded into the six-well plates at 50-60% confluence, and the constitutively-active Akt1 (caAkt1, S473D)-expressing adenovirus (from Dr. Li at Wenzhou Medical University (Zhang et al., 2016; Yang et al., 2020)) was added for 48h. After selection by puromycin, stable cells were established, and expression of caAkt1 was verified by Western blotting assays.

The nude mice xenograft assay. The athymic nude mice (half male, half female,5-6 week old and 18-19g weight) were provided by the SLAC Laboratory Animal Center (Shanghai, China). Mice were maintained under standard conditions, with 12-hour dark/12-hour light cycle, 24 ± 1°C temperatures, and free access of water and food. The primary human glioma cells P1 were trypsinized, washed and re-suspended. Cells (in 200 μL of Matrigel basic medium) were subcutaneously injected into the right armpit of the mice. Within three weeks of cell inoculation, the P1 glioma xenografts were established and tumor volume close to 100 mm^3. The xenograft-bearing nude mice then subject to intratumoral injection of adeno-associated virus (aav)-packed shRNA. The mice body weights and the tumor volumes (calculated through the described formula (Liu et al., 2018)) were measured every six days. For intracranial tumor implantation, the primary P1 human glioma cells were implanted using the previously described coordinates (Agnihotri et al., 2012). Magnetic resonance imaging (MRI) was utilized to visualize the tumor xenograft. On the day-25, all groups were sacrificed and tumors isolated through surgery. Tumor volumes were calculated by the described formula (Liu et al., 2018). The animal studies were approved by Institutional Animal Care and Use Committee (IACUC) and Ethics Committee of Soochow University.

Statistical analyses. Data were with normal distribution and were expressed as means ± standard deviation (SD). To examine statistical differences among multiple groups, one-way ANOVA followed by a Scheffe's f-test (SPSS 23.0, SPSS Co., Chicago, CA) was utilized. A two-tailed unpaired t test (Excel 2007) was applied to examine significance between two treatment groups. ***P*** values < 0.05 were considered statistically significant.

References

Agnihotri, S., Gajadhar, A.S., Ternamian, C., Gorlia, T., Diefes, K.L., Mischel, P.S., Kelly, J., McGown, G., Thorncroft, M., Carlson, B.L., *et al.* (2012). Alkylpurine-DNA-N-glycosylase confers resistance to temozolomide in xenograft models of glioblastoma multiforme and is associated with poor survival in patients. J Clin Invest 122, 253-266.

Bai, J.Y., Li, Y., Xue, G.H., Li, K.R., Zheng, Y.F., Zhang, Z.Q., Jiang, Q., Liu, Y.Y., Zhou, X.Z., and Cao, C. (2021). Requirement of Galphai1 and Galphai3 in interleukin-4-induced signaling, macrophage M2 polarization and allergic asthma response. Theranostics 11, 4894-4909.

Cai, S., Li, Y., Bai, J.Y., Zhang, Z.Q., Wang, Y., Qiao, Y.B., Zhou, X.Z., Yang, B., Tian, Y., and Cao, C. (2017). Galphai3 nuclear translocation causes irradiation resistance in human glioma cells. Oncotarget 8, 35061-35068.

Cao, C., Huang, X., Han, Y., Wan, Y., Birnbaumer, L., Feng, G.S., Marshall, J., Jiang, M., and Chu, W.M. (2009). Galpha(i1) and Galpha(i3) are required for epidermal growth factor-mediated activation of the Akt-mTORC1 pathway. Sci Signal 2, ra17.

Gao, Y.Y., Ling, Z.Y., Zhu, Y.R., Shi, C., Wang, Y., Zhang, X.Y., Zhang, Z.Q., Jiang, Q., Chen, M.B., Yang, S.F., *et al.* (2021). The histone acetyltransferase HBO1 functions as a novel oncogenic gene in osteosarcoma. Theranostics 11, 4599-4615.

Liang, J., Zhang, X.Y., Zhen, Y.F., Chen, C., Tan, H., Hu, J., and Tan, M.S. (2019). PGK1 depletion activates Nrf2 signaling to protect human osteoblasts from dexamethasone. Cell Death Dis 10, 888.

Liang, J.Q., Zhou, Z.T., Bo, L., Tan, H.N., Hu, J.H., and Tan, M.S. (2021). Phosphoglycerate kinase 1 silencing by a novel microRNA microRNA-4523 protects human osteoblasts from dexamethasone through activation of Nrf2 signaling cascade. Cell Death Dis 12, 964.

Liu, Y.Y., Chen, M.B., Cheng, L., Zhang, Z.Q., Yu, Z.Q., Jiang, Q., Chen, G., and Cao, C. (2018). microRNA-200a downregulation in human glioma leads to Galphai1 over-expression, Akt activation, and cell proliferation. Oncogene 37, 2890-2902.

Shan, H.J., Zhu, L.Q., Yao, C., Zhang, Z.Q., Liu, Y.Y., Jiang, Q., Zhou, X.Z., Wang, X.D., and Cao, C. (2021). MAFG-driven osteosarcoma cell progression is inhibited by a novel miRNA miR-4660. Mol Ther Nucleic Acids 24, 385-402.

Shao, N.Y., Wang, D.X., Wang, Y., Li, Y., Zhang, Z.Q., Jiang, Q., Luo, W., and Cao, C. (2018). MicroRNA-29a-3p Downregulation Causes Gab1 Upregulation to Promote Glioma Cell Proliferation. Cell Physiol Biochem 48, 450-460.

Sun, J., Huang, W., Yang, S.F., Zhang, X.P., Yu, Q., Zhang, Z.Q., Yao, J., Li, K.R., Jiang, Q., and Cao, C. (2018). Galphai1 and Galphai3mediate VEGF-induced VEGFR2 endocytosis, signaling and angiogenesis. Theranostics 8, 4695-4709.

Wang, Y., Liu, Y.Y., Chen, M.B., Cheng, K.W., Qi, L.N., Zhang, Z.Q., Peng, Y., Li, K.R., Liu, F., Chen, G., *et al.* (2021). Neuronal-driven glioma growth requires Galphai1 and Galphai3. Theranostics 11, 8535-8549.

Yang, H., Zhao, J., Zhao, M., Zhao, L., Zhou, L.N., Duan, Y., and Li, G. (2020). GDC-0349 inhibits non-small cell lung cancer cell growth. Cell Death Dis 11, 951.

Zhang, D., Xia, H., Zhang, W., and Fang, B. (2016). The anti-ovarian cancer activity by WYE-132, a mTORC1/2 dual inhibitor. Tumour Biol 37, 1327-1336.

Zhang, Y.M., Zhang, Z.Q., Liu, Y.Y., Zhou, X., Shi, X.H., Jiang, Q., Fan, D.L., and Cao, C. (2015). Requirement of Galphai1/3-Gab1 signaling complex for keratinocyte growth factor-induced PI3K-AKT-mTORC1 activation. J Invest Dermatol 135, 181-191.

Figure S1

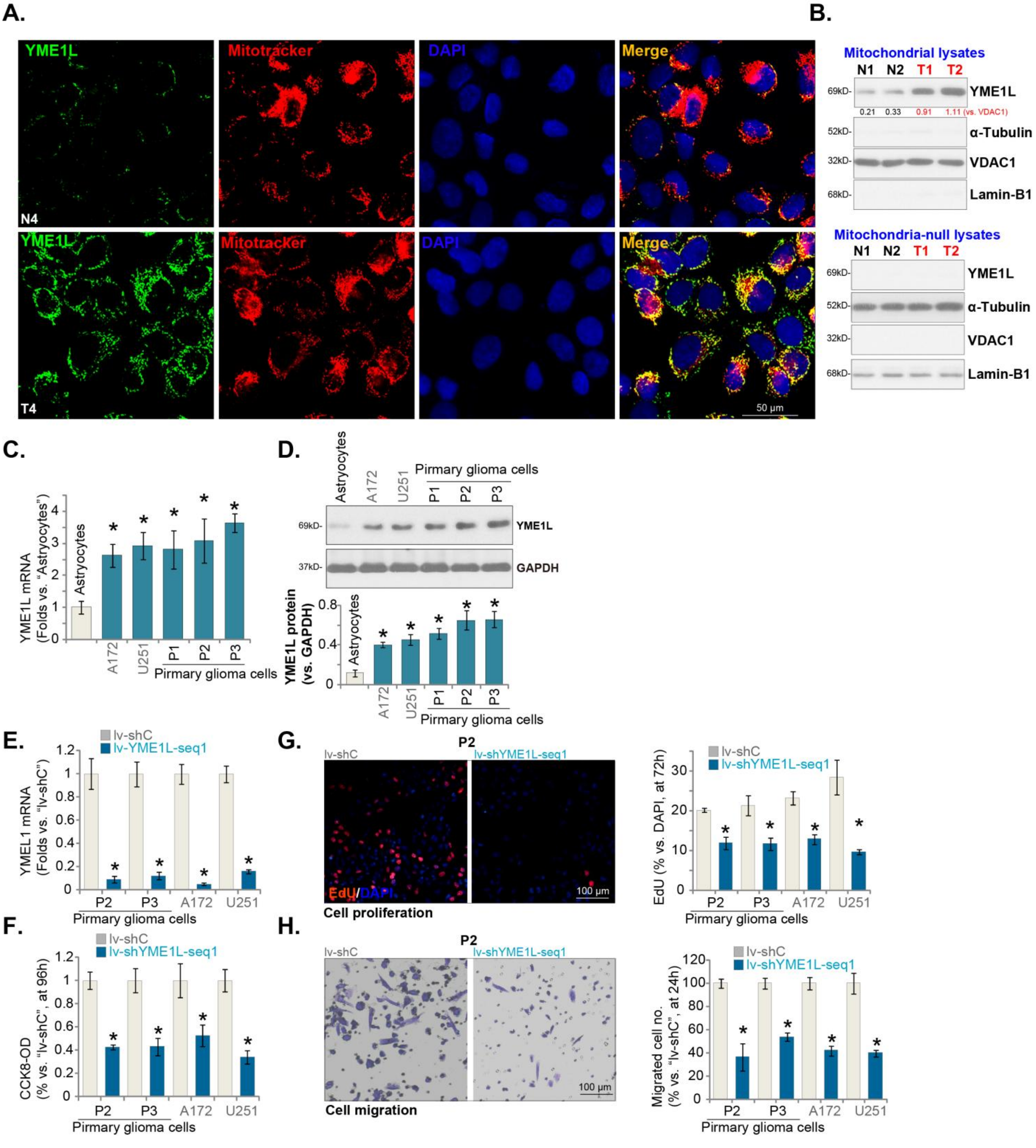

Figure S1. YME1L depletion leads to significant anti-glioma cell activity. The human tissue immuno-fluorescence images of YME1L (green fluorescence) and the MitoTracker red in the glioma slide and the adjacent normal brain slide of one representative glioma patient (Patient-3#) (**A**). Expression of listed proteins in mitochondrial lysates and mitochondria-null

lysates of two representative glioma patients (Patient 1# and 2#) was shown (**B**). Expression of *YME1L mRNA* (**C**) and protein (**D**) in the primary human astrocytes ("Astrocytes"), the immortalized (A172 and U251) and the primary (P2 and P3) human glioma cells was tested. The primary human glioma cells (P2 and P3, derived from different patients) or the immortalized cell lines (A172 and U251) cells, were infected with lv-shYME1L-seq1 or the lentiviral scramble shRNA ("lv-shC"), stable cells established after puromycin selection. *YME1L* mRNA expression was tested by qRT-PCR assays (**E**); Cells were further cultured for applied time periods, cell viability, proliferation and migration were tested by CCK-8 (**F**), nuclear EdU staining (**G**) and "Transwell" (**H**) assays, respectively. The data were presented as mean ± standard deviation (SD, n = 5). * $P < 0.05$ versus "Astrocytes"/"lv-shC" cells. The experiments were repeated five times with similar results obtained. Scale bar = 50 μm (**A**). Scale bar = 100 μm (**G** and **H**).

Figure S2

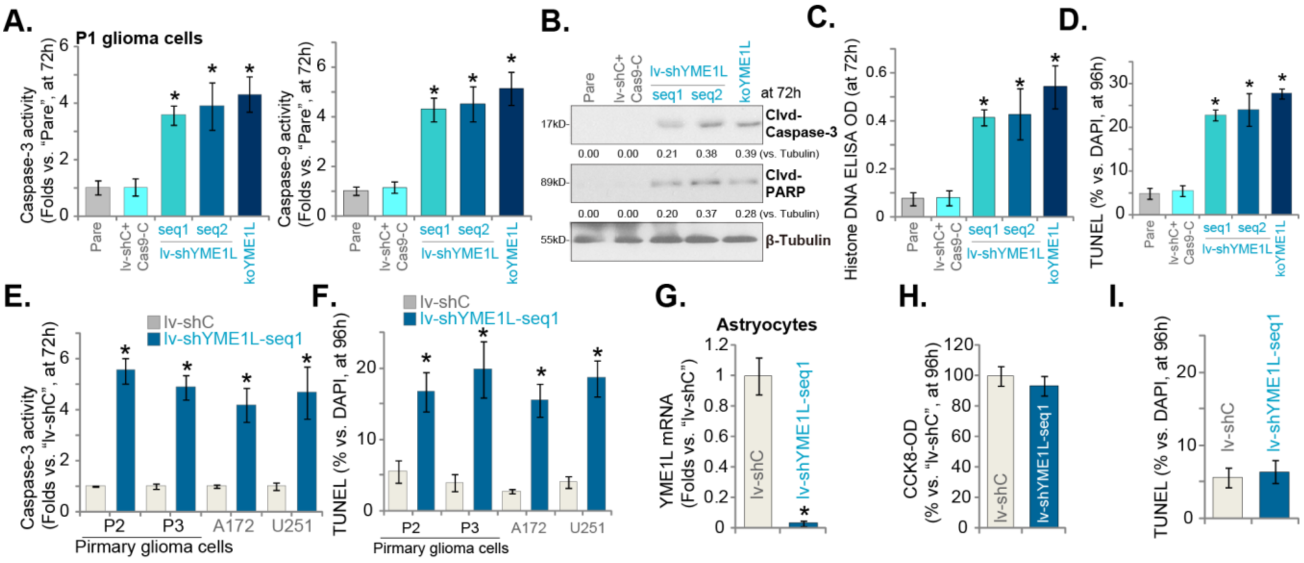

Figure S2. YME1L depletion leads to apoptosis activation in glioma cells. The P1 primary human glioma cells, stably expressing the applied YME1L shRNA (lv-shYME1L-seq1/2, two different sequences) or the lenti-CRSIPR/Cas9-YME1L-KO-puro construct ("koYME1L"), were established. Control P1 glioma cells were transduced with the lentiviral scramble shRNA plus the CRSIPR/Cas9 empty vector ("lv-shC+Cas9-C"). Cells were further cultured for applied time periods, the caspase-3 activity and the caspase-9 activity were tested (**A**); Expression of listed proteins in total cell lysates was tested by Western blotting assays (**B**), and Histone-bound DNA contents examined by the ELISA method (**C**); Cell apoptosis was examined by recording nuclear TUNEL ratio (**D**). The primary human glioma cells (P2 and P3, derived from different patients) or the immortalized cell lines (A172 and U251) cells, were infected with lv-shYME1L-seq1 or the lentiviral scramble shRNA ("lv-shC"), stable cells established after puromycin selection and cells cultured for applied time periods; The caspase-3 activity (**E**) and cell apoptosis (by recording TUNEL-nuclei ratio, **F**) were tested similarly. The primary human astrocytes ("Astrocytes") were infected with lv-shYME1L-seq1 or the lentiviral scramble shRNA ("lv-shC"), stable cells established after puromycin selection, expression of *YME1L* mRNA was tested (**G**); Cells were cultured for applied time periods, cell viability (**H**) and apoptosis (**I**) were tested as well. "Pare" stands for the parental control cells. The data were presented as mean ± standard deviation (SD, n = 5). * ***P*** < 0.05 versus "Pare"/"lv-shC" cells. The experiments were repeated five times with similar results obtained.

Figure S3

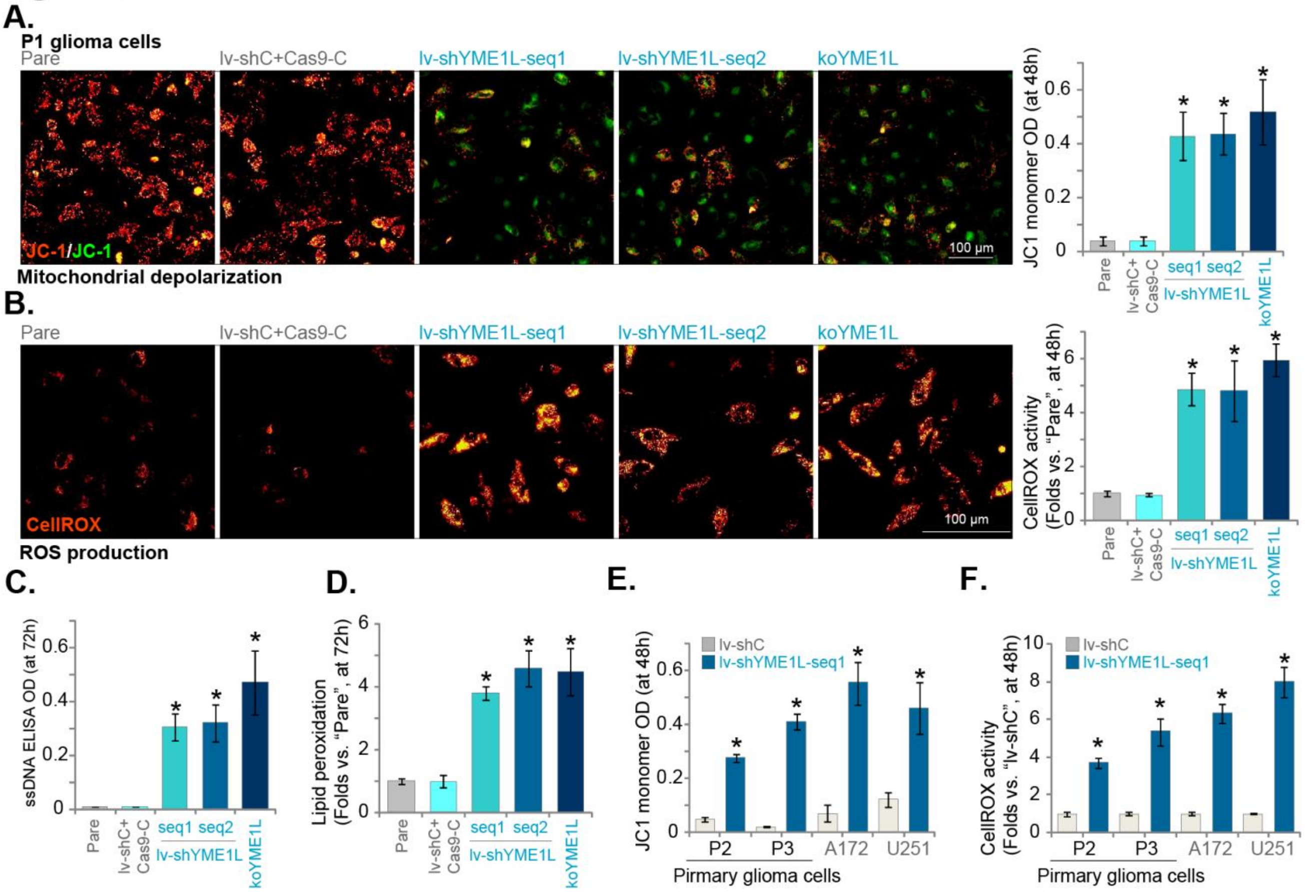

Figure S3. YME1L depletion disrupts mitochondrial functions in glioma cells. The P1 primary human glioma cells, stably expressing the applied YME1L shRNA (lv-shYME1L-seq1/2, two different sequences) or the lenti-CRSIPR/Cas9-YME1L-KO-puro construct ("koYME1L"), were established. Control P1 glioma cells were transduced with the lentiviral scramble shRNA plus the CRSIPR/Cas9 empty vector ("lv-shC+Cas9-C"). Cells were further cultured for applied time periods, mitochondrial depolarization and ROS production were tested by JC-1 (**A**) and CellROX (**B**) staining assays, respectively; The single strand DNA (ssDNA) contents (ELISA OD, **C**) and the lipid peroxidation intensity (by testing TBAR activity, **D**) were examined as well. The primary human glioma cells (P2 and P3) or the immortalized cell lines (A172 and U251) cells, were infected with lv-shYME1L-seq1 or the lentiviral scramble shRNA ("lv-shC"), stable cells established after puromycin selection and cultured for applied time periods, mitochondrial depolarization (by testing JC-1 green monomer intensity, **E**) and ROS production (by measuring the CellROX red fluorescence intensity, **F**) were tested similarly. "Pare" stands for the parental control cells. The data were presented as mean ± standard deviation (SD, n = 5). * $P < 0.05$ versus "Pare"/"lv-shC" cells. The experiments were repeated five times with similar results obtained. Scale bar = 100 μm (**A** and **B**).

Figure S4

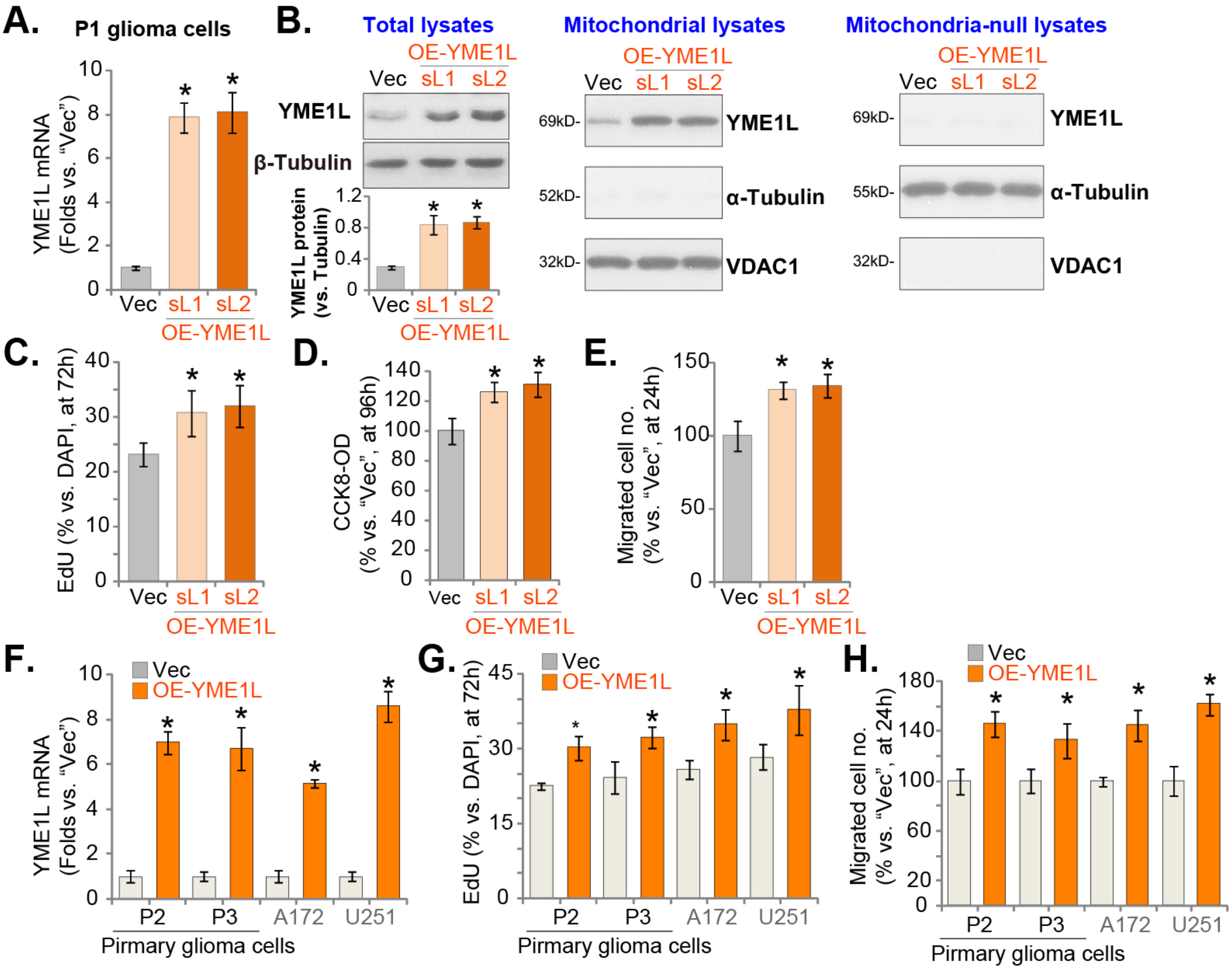

Figure S4. YME1L overexpression accelerates glioma cell growth and proliferation. The primary human glioma cells (P1, P2 or P3) or the immortalized cell lines (A172 and U251) cells, were infected with the lentivirus encoding the wild-type YME1L cDNA ("OE-YME1L") or the empty vector ("Vec"), stable cells established after puromycin selection. Expression of *YME1L* mRNA and listed proteins (in different lysates) was tested (**A**, **B**, **F**); Cells were further cultured for applied time periods, cell proliferation, viability, migration were tested by the nuclear EdU staining (**C** and **G**), CCK-8 (**D**) and "Transwell" (**E** and **H**) assays, respectively, with results quantified. The data were presented as mean ± standard deviation (SD, n = 5). * ***P*** < 0.05 versus "Vec" cells. The experiments were repeated five times with similar results obtained.

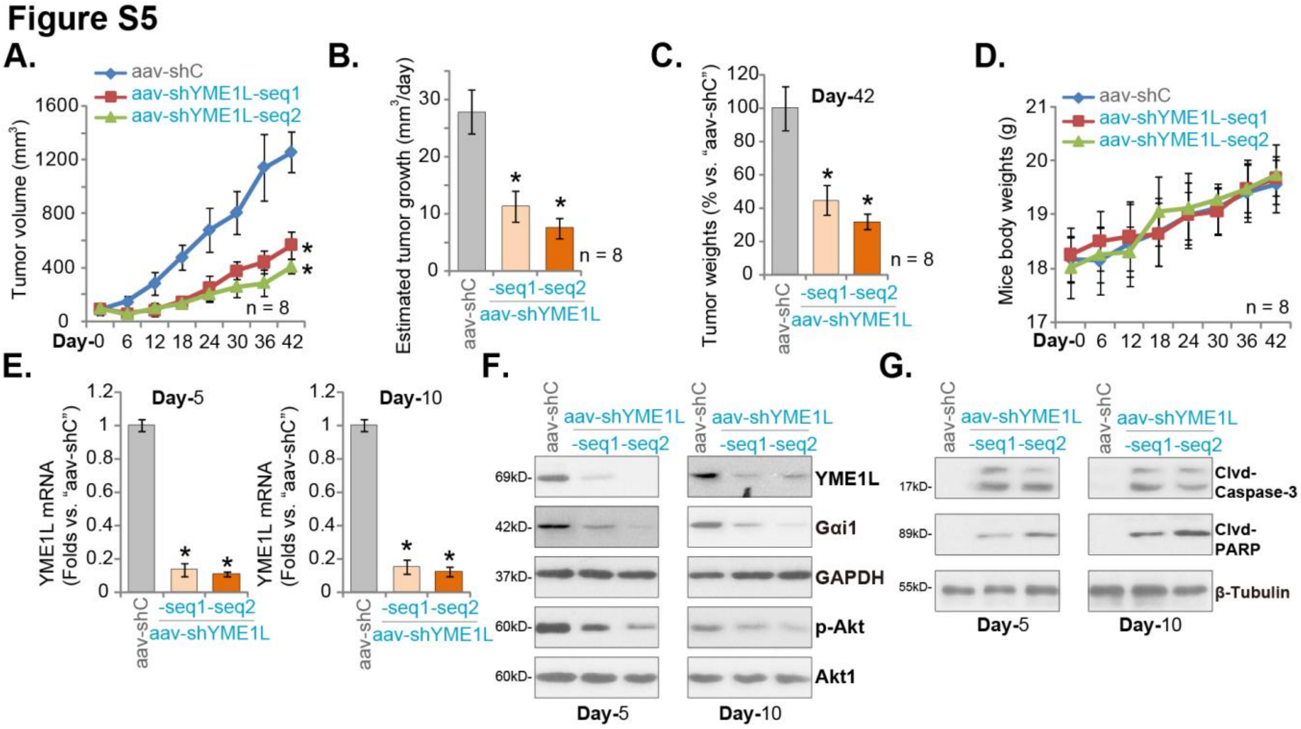

Figure S5. YME1L depletion inhibits subcutaneous and orthotopic growth of primary glioma xenografts in nude mice. The nude mice bearing subcutaneous P1 glioma xenografts were subject to intratumoral injection of designated aav-packed shRNA (aav-shYME1L-seq1, aav-shYME1L-seq2 or aav-shC), virus injection was carried out daily for 14 consecutive days ("Day-0" to "Day-14"); The xenograft volumes (**A**) and mice body weights (**D**) were recorded every six days ("Day-0" to "Day-42"). The estimated daily xenograft growth was calculated as described (**C**). At "Day-42", all xenograft tumors were isolated and weighted (**C**). At experimental "Day-5" and "Day-10", three hours after aav injection, one xenograft of each group was isolated and fresh xenograft lysates were obtained. Expression of listed genes was tested by qRT-PCR (**E**) and Western blotting (**F** and **G**) assays. * $\boldsymbol{P} < 0.05$ versus "aav-shC" groups.
